# Associations of Hemoglobin Glycation Index with Glucose Variability and Time in Ranges in Patients with Type 2 Diabetes Undergoing Continuous Glucose Monitoring

**DOI:** 10.7150/ijms.129813

**Published:** 2026-08-11

**Authors:** Xiu-Yu Wei, Jun-Sing Wang

**Affiliations:** 1 Division of Endocrinology and Metabolism, Department of Internal Medicine, Taichung Veterans General Hospital, Taichung, Taiwan.; 2 Department of Medicine, School of Medicine, National Yang Ming Chiao Tung University, Taipei, Taiwan.; 3 Department of Post-Baccalaureate Medicine, College of Medicine, National Chung Hsing University, Taichung, Taiwan.; 4 Department of Music, Tunghai University, Taichung, Taiwan.

**Keywords:** continuous glucose monitoring, glycated hemoglobin, hemoglobin glycation index, type 2 diabetes

## Abstract

**Background:**

Discordance between glycated hemoglobin (HbA1c) levels and blood glucose is commonly observed in clinical practice. The hemoglobin glycation index (HGI) has been proposed as a measure to quantify individual variations in glycation. Our study aimed to investigate the association between HGI and glucose variability assessed by continuous glucose monitoring (CGM) in patients with type 2 diabetes (T2D) treated with metformin monotherapy.

**Methods:**

Adults with T2D treated with metformin monotherapy (daily dose ≥ 1500 mg) who had HbA1c levels ≥ 7.0% (n = 100; mean age 54.0 ± 8.2 years; 52.0% female) undergoing CGM were analyzed. Predicted HbA1c was derived from linear regression of HbA1c on fasting plasma glucose, and the HGI was calculated as observed HbA1c minus predicted HbA1c. CGM metrics—including standard deviation, coefficient of variation, mean amplitude of glycemic excursions (MAGE), and time in ranges—were analyzed to evaluate their associations with HGI.

**Results:**

HGI was significantly associated with mean sensor glucose (β = 13.60, 95% CI 5.16-22.04, p = 0.002) and CGM-derived glucose variability metrics, including standard deviation (β = 7.53, 95% CI 4.49-10.58, p < 0.001), coefficient of variation (β = 2.22, 95% CI 0.29-4.15, p = 0.025), and MAGE (β = 22.14, 95% CI 14.59-29.69, p < 0.001). These associations remained significant after multivariable adjustment. HGI was also negatively associated with time in range (β = -7.29, 95% CI -12.79 to -1.79, p = 0.010) and positively associated with time above range (β = 7.35, 95% CI 1.69-13.01, p = 0.011).

**Conclusion:**

These findings highlight a significant association between HGI and glucose variability from CGM, suggesting that HGI may serve as a complementary marker in diabetes management.

## Introduction

Glycated hemoglobin (HbA1c) remains the most widely accepted indicator of long-term glycemic control in diabetes management 1. Compared with fasting or 2-h post-load plasma glucose measurements, HbA1c provides greater analytical stability and reproducibility, making it a practical tool for both clinical and research settings. However, discrepancies between HbA1c and other measures of glycemia have been repeatedly documented 2-5, suggesting that HbA1c does not fully capture the complexity of glucose dynamics.

Interindividual differences in HbA1c levels among individuals with similar mean glucose concentrations have been consistently observed, and these differences tend to persist over time 6-8. To describe this phenomenon, the hemoglobin glycation index (HGI) is proposed 7, defined as the deviation of an individual's measured HbA1c from a predicted value derived from the population-level relationship between fasting plasma glucose (FPG) and HbA1c 9. Elevated HGI has been linked to adverse cardiovascular outcomes and mortality 10,11, underscoring the importance of identifying factors that influence its variability.

Although the precise mechanisms underlying variations in the HbA1c-glucose relationship remain uncertain, both analytical and biological factors, along with physiological and demographic characteristics, are believed to contribute 6-8,12-17. It has been postulated that individuals with higher HGI may experience unrecognized postprandial or transient hyperglycemia that fasting glucose or self-monitoring cannot capture 10. With the advancement and widespread use of continuous glucose monitoring (CGM), it has become possible to capture detailed patterns of glycemic fluctuations throughout the day—beyond what can be reflected by HbA1c alone. Since the HGI may be influenced by various aspects of glycemic variability, our study investigated the associations between HGI and CGM-derived measures of glucose fluctuations, including the standard deviation and coefficient of variation of sensor glucose and the mean amplitude of glycemic excursions (MAGE), in patients with type 2 diabetes (T2D). Because both hyperglycemia and hypoglycemia reflect glucose fluctuations, we also assessed the associations between HGI and CGM-derived time-in-range metrics.

## Materials and Methods

### Study population and ethics

We retrospectively analyzed CGM data from outpatients with T2D. This study was approved by the Institutional Review Board of Taichung Veterans General Hospital, Taichung, Taiwan (approval number CE25596B, approved on August 13, 2025) and conducted in accordance with the Declaration of Helsinki. The Institutional Review Board granted a waiver of informed consent due to the retrospective study design and use of de-identified data. Eligible study subjects were outpatients with T2D who had received metformin monotherapy, had an HbA1c ≥ 7.0%, and had undergone ambulatory CGM. Demographic characteristics, FPG, and HbA1c levels were obtained from electronic medical records. All data were de-identified prior to analysis.

### Variable selection

All patients underwent CGM using the Medtronic MiniMed Continuous Glucose Monitoring System (Northridge, CA, USA). FPG and HbA1c values obtained on the first day of CGM were used to calculate the HGI. Several CGM metrics 18 were used for analyses, including mean sensor glucose, time in range (TIR, 70-180 mg/dL), time above range (TAR, > 180 mg/dL), and time below range (TBR, < 70 mg/dL). Metrics for glucose variability included standard deviation and coefficient of variation of sensor glucose, and MAGE 19. We chose MAGE as a measure of glucose variability because it has been associated with oxidative stress 20 and adverse cardiovascular outcomes 21.

FPG and HbA1c values from a single visit on the first day of CGM were used to calculate HGI-FPG 9. Briefly, predicted HbA1c was estimated from FPG using an equation derived from a Taiwanese population with T2D: predicted HbA1c (%) = FPG (mg/dL) × 0.0225 + 4.3806 9. HGI was defined as the difference between the observed and predicted HbA1c (HGI = observed HbA1c - predicted HbA1c). For example, for a patient with an FPG of 150 mg/dL and an HbA1c of 8.0%, the predicted HbA1c would be 7.76%, yielding an HGI-FPG of 0.24%. For comparison, estimated HbA1c derived from CGM (glucose management indicator, GMI) was used to determine HGI-GMI.

### Statistical analysis

All statistical analyses were performed using the Statistical Package for the Social Sciences (SPSS, version 22.0; IBM Corp., Armonk, NY, USA). Categorical variables are presented as numbers (percentages) and continuous variables as mean ± standard deviation. Associations between HGI and CGM-derived metrics were assessed using linear regression analysis, adjusted for age, sex, body mass index, and duration of diabetes. A two-sided p-value < 0.05 was considered statistically significant.

## Results

A total of 100 patients with T2D who had an HbA1c ≥ 7.0% on metformin monotherapy (daily dose ≥ 1500 mg) were included in our analyses. The characteristics of study patients are shown in Table 1. These patients had suboptimal glycemic control (mean FPG 142.5 ± 37.5 mg/dL, mean HbA1c 7.9 ± 1.2 %). The mean predicted HbA1c according to their FPG was 7.6 ± 0.8 %, while the mean HGI-FPG was 0.3 ± 0.9 %. Table 2 shows metrics derived from the CGM. The mean sensor glucose was 159.0 ± 40.5 mg/dL, and the GMI was 7.1 ± 1.0 %, indicating suboptimal glycemic control in the study population. The mean HGI-GMI was 0.8 ± 0.7 %. The TIR, TAR, and TBR were 69.3 ± 26.2 %, 29.1 ± 26.9 %, and 1.6 ± 3.6 %, respectively.

**Figure 1:**
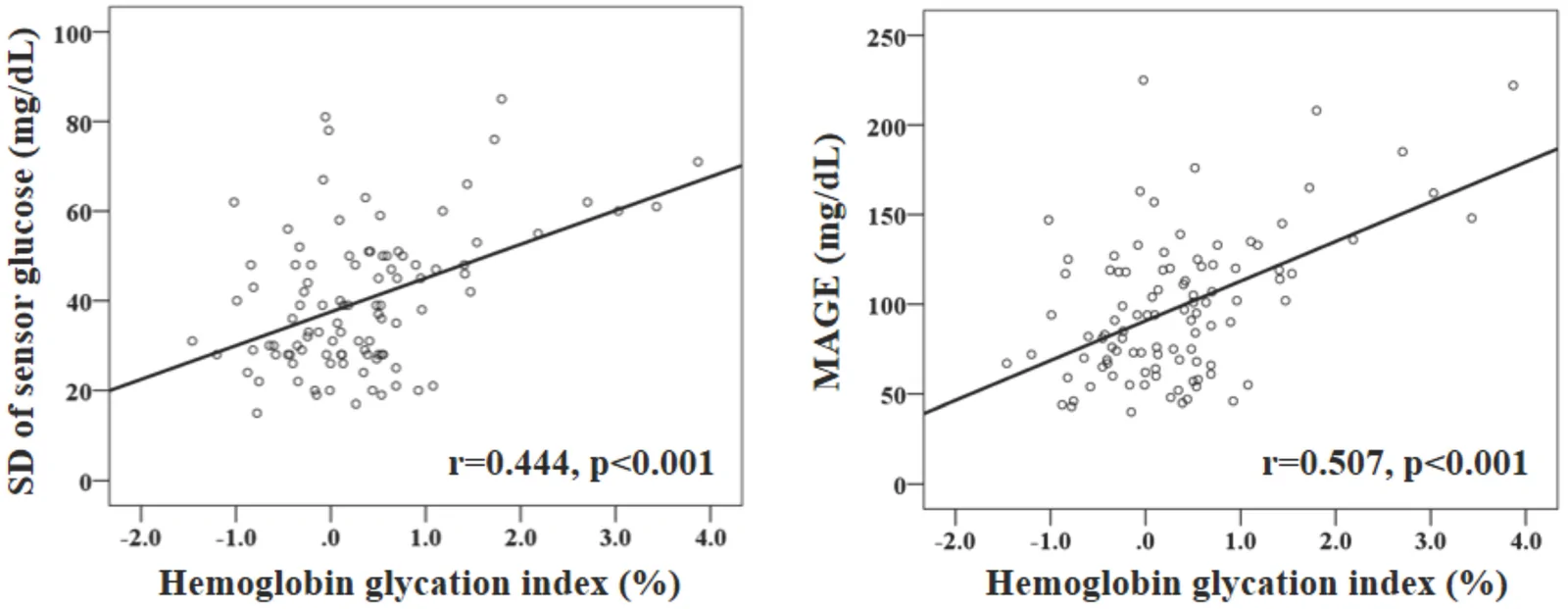
Correlations of hemoglobin glycation index with standard deviation (SD) of sensor glucose (left panel) and mean amplitude of glycemic excursions (right panel). MAGE, mean amplitude of glycemic excursions. The hemoglobin glycation index was defined as the difference between the observed HbA1c and the predicted HbA1c based on fasting plasma glucose. The MAGE is a continuous glucose monitoring-derived metric of glycemic variability that quantifies the average amplitude of major glucose fluctuations, defined as glucose excursions exceeding one SD of all sensor glucose values.

Figure 1 shows the correlations of HGI-FPG with standard deviation of sensor glucose (r = 0.444, p < 0.001) and MAGE (r = 0.507, p < 0.001). The associations of HGI-FPG with CGM metrics of glucose variability were examined in Table 3. We found that HGI-FPG was significantly associated with mean sensor glucose (β coefficient 13.60, 95% CI 5.16 to 22.04, p = 0.002) and several metrics of glucose variability, including standard deviation (β coefficient 7.53, 95% CI 4.49 to 10.58, p < 0.001) and coefficient of variation (β coefficient 2.22, 95% CI 0.29 to 4.15, p = 0.025) of sensor glucose, and MAGE (β coefficient 22.14, 95% CI 14.59 to 29.69, p < 0.001). The aforementioned associations remained significant after multivariate adjustment (all p < 0.05).

Table 4 shows the associations of HGI-GMI with several CGM metrics. HGI-GMI was not associated with mean sensor glucose. The associations of HGI-GMI with CGM-derived metrics of glucose variability were comparable to those observed for HGI-FPG. Table 5 shows the associations of HGI-FPG with TIR, TAR, and TBR. HGI-FPG was negatively associated with TIR (β coefficient -7.29, 95% CI -12.79 to -1.79, p = 0.010) and positively associated with TAR (β coefficient 7.35, 95% CI 1.69 to 13.01, p = 0.011). These associations remained significant after multivariate adjustment. Nevertheless, HGI-FPG was not associated with TBR (β coefficient -0.06, 95% CI -1.02 to 0.90, p = 0.899).

Table 6 shows time in ranges according to HGI-FPG in patients with HbA1c ≥ 7.0% and < 7.0%. Among patients with HbA1c ≥ 7.0%, those with higher HGI-FPG (> 0% vs ≤ 0%) had lower FPG (142.1 ± 33.5 vs 165.3 ± 42.1 mg/dL, p = 0.010) but higher HbA1c (8.4 ± 1.2 vs 7.7 ± 0.8 %, p = 0.009). There was no significant between-group differences in TIR, TAR, and TBR. Nevertheless, it is noteworthy that patients with HbA1c ≥ 7.0% and higher HGI-FPG (> 0% vs ≤ 0%) exhibited approximately 50% greater TBR (1.5 ± 4.2 vs. 1.0 ± 2.1 %, p = 0.611). Similar findings were observed in patients with HbA1c < 7.0%. Notably, those with higher HGI-FPG (> 0% vs ≤ 0%) had a fourfold greater TBR (7.6 ± 2.3 vs. 1.9 ± 2.7 %, p = 0.013) at comparable HbA1c levels (6.6 ± 0.4 vs 6.5 ± 0.3 %, p = 0.811) compared with patients with lower HGI-FPG.

## Discussion

Our findings indicate that the HGI-FPG is significantly associated with increased TAR in patients with T2D under Metformin monotherapy. HGI represents the gap between a person's actual HbA1c value and the level estimated from their blood glucose concentration using a regression model. In earlier studies, the predicted HbA1c has been commonly derived from FPG or mean blood glucose obtained through self-monitoring or CGM techniques 6-8,12. While analytical variation in HbA1c, FPG, or mean blood glucose (MBG) may partly explain discrepancies in HGI, individual biological differences—such as genetic and environmental influences—also contribute to variability across the population 13. Several physiological and demographic factors have been linked to HGI, including sex 14,15, ethnicity 16,17, obesity, systemic inflammation, iron metabolism, fructosamine-3-kinase activity, glucose transport across red blood cells, intracellular pH, levels of 2,3-bisphosphoglycerate, and postprandial glycemia 13.

A previous study, Joung HN et al. 22 investigated the glycation gap, fasting blood glucose-based HGI, and MBG-based HGI in patients with T2D using 72-hour CGM. The study demonstrated that both the glycation gap and HGI were highly reproducible within individuals, and that fasting blood glucose-based and MBG-based HGIs were positively correlated. These findings supported the concept that HGI reflected interindividual differences in hemoglobin glycation rate or red blood cell characteristics rather than being an analytical artifact caused by glucose measurement methods. However, the study made limited use of the rich data available from CGM, focusing primarily on MBG as the predictive variable for HbA1c, without evaluating other key CGM-derived parameters such as glycemic variability, diurnal glucose fluctuation, or time-in-range metrics. These dynamic glucose features might better capture temporal exposure to glucose and the oscillatory nature of glycemia, which could influence the glycation process itself. Therefore, the omission of these CGM-derived metrics might underestimate the contribution of glucose dynamics to the HGI and glycation gap. In contrast, our findings demonstrate that HGI calculated from FPG (HGI-FPG) was positively correlated not only with MBG, but also with multiple indices of glucose variability obtained from CGM, including standard deviation, coefficient of variation, and MAGE (Table 3). These findings suggest that individuals with high HGI may experience significant postprandial or within-day glycemic excursions that is not adequately reflected by FPG alone.

The associations of HGI-GMI with CGM-derived metrics of glucose variability were comparable to those observed for HGI-FPG, however, HGI-GMI was not associated with mean sensor glucose (Table 4). HGI-GMI and HGI-FPG represent different aspects of “glycation gap”. HGI-GMI represents the difference between “mean sensor glucose [estimated HbA1c]” and “observed HbA1c”, which likely reflects the “biological glycation gap” 23. In contrast, HGI-FPG represents the difference between “HbA1c estimated from FPG” and “observed HbA1c”. Because FPG does not capture daytime hyperglycemia (e.g., postprandial excursions), HGI-FPG likely reflects glucose dynamics not captured by FPG 24. The aforementioned considerations may explain our findings that HGI-FPG was associated with mean sensor glucose (largely influenced by daytime hyperglycemia) (Table 3), whereas HGI-GMI was not, as it reflects the biological glycation gap (Table 4). Similarly, HGI-FPG was negatively associated with TIR and positively associated with TAR (Table 5), whereas HGI-GMI was not (p=0.674 and 0.559, respectively; data not shown in the Results). Taken together, HGI-FPG may help identify patients who are more likely to have high glucose variability, which is often overlooked without multiple self-monitoring blood glucose measurements or CGM in patients with T2D.

Notably, in our study, a higher HGI-FPG is significantly associated with increased TAR, even after adjusting for potential confounders such as age, sex, body mass index, and duration of diabetes (Table 5). This finding suggests that HGI may be a meaningful predictor of glycemic control beyond traditional markers. Previous studies have reported that a higher HGI is associated with greater postprandial glycemic excursions and elevated 2-hour plasma glucose levels following an oral glucose tolerance test, both of which are known contributors to increased TAR 25,26. These postprandial fluctuations are not fully captured by FPG, which is commonly used in the calculation of HGI. As such, the rationale for our hypothesis lies in the limitation of using FPG-derived HbA1c alone, which overlooks the substantial contribution of postprandial glucose excursions to HbA1c variability. Moreover, postprandial hyperglycemia and greater glycemic variability are known to induce prolonged periods of hyperglycemia, thereby increasing TAR and promoting non-enzymatic glycation of hemoglobin 27,28. Despite this biological plausibility, studies directly linking HGI with CGM-derived TAR remain limited. Our findings therefore contribute novel evidence to support the utility of HGI-FPG as a marker of glycemic variability and postprandial dysregulation.

Another notable finding of our study is that within a given HbA1c range (≥ 7.0% or < 7.0%), patients with higher HGI-FPG exhibited lower fasting glucose, but higher HbA1c and greater TBR (Table 6). This observation is consistent with several previous reports 10,29 linking higher HGI values to an increased risk of hypoglycemia. In the ACCORD trial 10, participants with elevated HGI experienced more frequent hypoglycemic events across treatment arms, and this risk was further amplified under intensive glucose-lowering therapy. Similarly, Bei-Si Lin et al. 29 found that a higher HGI was significantly associated with a greater incidence of hypoglycemia, even among individuals who maintained a TIR within the target range. The non-significant association between HGI-FPG and TBR in our overall study population (Table 5) may be explained by the fact that all patients were treated exclusively with metformin, which is known to carry a low risk of hypoglycemia. Although we did not observe any hospitalizations for hypoglycemia during the study period, it is noteworthy that even asymptomatic hypoglycemia may have detrimental effects on the cardiovascular system 30. Taken together, the associations between HGI-FPG and TBR within a given HbA1c range, as well as glucose variability metrics, appear to be clinically relevant.

In recent decades, CGM technology has gained increasing popularity and provides a wide range of metrics for assessing glycemic control. Among these, the HGI has been linked to various clinical outcomes, including microvascular complications 31,32, cardiovascular events 10,28, and the degree of HbA1c reduction following treatment interventions 33. Our findings further emphasize the clinical relevance of HGI by highlighting its association with increased TAR and greater glycemic variability. These results underscore the importance of incorporating TAR into individualized treatment strategies for patients with T2D, alongside efforts to improve the TIR to achieve optimal glycemic control and reduce the risk of diabetes-related complications.

The strength of our study is that the association between HGI and glucose variability were investigated using CGM data, which offered an ambulatory glucose profile. Nevertheless, this study has several limitations that warrant consideration. One, the sample size is relatively small. Future work involving more extensive patient populations is essential to validate and extend our observations. The other, the eligibility criteria of this study may limit the generalizability of our results, and our findings should be validated in diabetic patients receiving a variety of glucose-lowering treatments. It is noteworthy that metformin treatment has been independently associated with the glycation gap 34, however, data on HGI before and after metformin treatment were not available in our study. Further investigations are warranted to clarify the effects of different glucose-lowering therapies on HGI in patients with T2D.

## Conclusion

Our study demonstrates that HGI was associated with glucose fluctuations, TIR, and TAR among people with T2D. Given the limitations of HbA1c in reflecting glycemic variability, HGI may help identify patients with T2D who are likely to have fluctuations in ambulatory glucose profiles.

## Figures and Tables

**Table 1:** Characteristics of study population

Number of patients	100
Age, years	54.0 ± 8.2
Female, n (%)	52 (52.0)
Body mass index, kg/m^2^	25.5 ± 3.5
Duration of diabetes, years	7.3 ± 4.6
FPG, mg/dl	142.5 ± 37.5
Observed HbA1c, %	7.9 ± 1.2
Predicted HbA1c according to FPG, %	7.6 ± 0.8
HGI-FPG, %	0.3 ± 0.9

Data are presented as mean ± SD or numbers (percentages). FPG, fasting plasma glucose. HbA1c, glycated hemoglobin. HGI, hemoglobin glycation index. HGI-FPG, HGI defined as the difference between the observed HbA1c and the predicted HbA1c based on FPG.

**Table 2:** Metrics from continuous glucose monitoring (CGM) of the study population

Mean sensor glucose values, mg/dl	159.0 ± 40.5
Glucose management indicator (GMI), %	7.1 ± 1.0
Hemoglobin glycation index (HGI)-GMI, %	0.8 ± 0.7
SD of sensor glucose values, mg/dl	40.0 ± 15.5
Coefficient of variation of sensor glucose values, %	25.5 ± 9.0
Mean amplitude of glycemic excursions (MAGE), mg/dl	97.9 ± 40.0
Time in range (TIR) (70-180 mg/dl), %	69.3 ± 26.2
Time above range (TAR) (> 180 mg/dl), %	29.1 ± 26.9
Time below range (TBR) (< 70 mg/dl), %	1.6 ± 3.6

Data are presented as mean ± SD. GMI, an estimate of HbA1c based on the mean glucose measured by CGM. HGI-GMI, HGI calculated as the difference between the observed HbA1c and the GMI. Coefficient of variation of sensor glucose values, a CGM-derived measure of glycemic variability calculated as (SD of sensor glucose / mean sensor glucose) × 100%. MAGE, a CGM-derived metric of glycemic variability that quantifies the average amplitude of major glucose fluctuations, defined as glucose excursions exceeding one SD of all sensor glucose values.

**Table 3:** Associations of HGI-FPG with mean sensor glucose and CGM metrics of glucose variability

Dependent variable: mean sensor glucose	β coefficient (95% CI)	P
Model 1	13.60 (5.16, 22.04)	0.002
Model 2	14.09 (5.10, 23.07)	0.002
Model 3	14.34 (5.21, 23.46)	0.002
Dependent variable: Standard deviation of sensor glucose	β coefficient (95% CI)	P
Model 1	7.53 (4.49, 10.58)	< 0.001
Model 2	7.96 (4.72, 11.20)	< 0.001
Model 3	7.86 (4.58, 11.14)	< 0.001
Dependent variable: Coefficient of variation of sensor glucose	β coefficient (95% CI)	P
Model 1	2.22 (0.29, 4.15)	0.025
Model 2	2.39 (0.33, 4.44)	0.023
Model 3	2.29 (0.21, 4.63)	0.032
Dependent variable: Mean amplitude of glycemic excursions	β coefficient (95% CI)	P
Model 1	22.14 (14.59, 29.69)	< 0.001
Model 2	23.55 (15.55, 31.55)	< 0.001
Model 3	23.13 (15.04, 31.21)	< 0.001

CGM, continuous glucose monitoring. FPG, fasting plasma glucose. HGI, hemoglobin glycation index. HGI-FPG, HGI defined as the difference between the observed HbA1c and the predicted HbA1c based on FPG. Coefficient of variation of sensor glucose values, a CGM-derived measure of glycemic variability. Mean amplitude of glycemic excursions, a CGM-derived metric of glycemic variability that quantifies the average amplitude of major glucose fluctuations. Model 1, unadjusted. Model 2, adjusted for age and sex. Model 3, adjusted for variables in Model 2 plus body mass index and diabetes duration.

**Table 4:** Associations of HGI-GMI with CGM mean sensor glucose and metrics of glucose variability

Dependent variable: mean sensor glucose	β coefficient (95% CI)	P
Model 1	-1.57 (-12.54, 9.41)	0.778
Model 2	-2.39 (-13.62, 8.83)	0.673
Model 3	-2.31 (-13.75, 9.13)	0.690
Dependent variable: Standard deviation of sensor glucose	β coefficient (95% CI)	P
Model 1	5.04 (0.96, 9.12)	0.016
Model 2	4.95 (0.76, 9.14)	0.021
Model 3	5.06 (0.82, 9.30)	0.020
Dependent variable: Coefficient of variation of sensor glucose	β coefficient (95% CI)	P
Model 1	3.19 (0.82, 5.56)	0.009
Model 2	3.26 (0.84, 5.69)	0.009
Model 3	3.28 (0.83, 5.74)	0.009
Dependent variable: Mean amplitude of glycemic excursions	β coefficient (95% CI)	P
Model 1	17.80 (7.57, 28.04)	0.001
Model 2	17.73 (7.23, 28.23)	0.001
Model 3	17.91 (7.33, 28.48)	0.001

CGM, continuous glucose monitoring. GMI, glucose management indicator. HGI, hemoglobin glycation index. GMI, an estimate of HbA1c based on the mean glucose measured by CGM. HGI-GMI, HGI calculated as the difference between the observed HbA1c and the GMI. Coefficient of variation of sensor glucose values, a CGM-derived measure of glycemic variability. Mean amplitude of glycemic excursions, a CGM-derived metric of glycemic variability that quantifies the average amplitude of major glucose fluctuations. Model 1, unadjusted. Model 2, adjusted for age and sex. Model 3, adjusted for variables in Model 2 plus body mass index and diabetes duration.

**Table 5:** Associations of HGI-FPG with time in ranges from CGM

Dependent variable: Time in range (TIR)	β coefficient (95% CI)	P
Model 1	-7.29 (-12.79, -1.79)	0.010
Model 2	-7.58 (-13.41, -1.75)	0.011
Model 3	-7.62 (-13.56, -1.69)	0.012
Dependent variable: Time above range (TAR)	β coefficient (95% CI)	P
Model 1	7.35 (1.69, 13.01)	0.011
Model 2	7.71 (1.70, 13.71)	0.012
Model 3	7.74 (1.63, 13.84)	0.014
Dependent variable: Time below range (TBR)	β coefficient (95% CI)	P
Model 1	-0.06 (-1.02, 0.90)	0.899
Model 2	-0.13 (-1.15, 0.89)	0.805
Model 3	-0.11 (-1.15, 0.92)	0.829

CGM, continuous glucose monitoring. FPG, fasting plasma glucose. HGI, hemoglobin glycation index. HGI-FPG, HGI defined as the difference between the observed HbA1c and the predicted HbA1c based on FPG. TIR, the percentage of time that glucose levels are within the target range (70-180 mg/dL). TAR, the percentage of time that glucose levels are above the target range (> 180 mg/dL). TBR, the percentage of time that glucose levels are below the target range (< 70 mg/dL). Model 1, unadjusted. Model 2, adjusted for age and sex. Model 3, adjusted for variables in Model 2 plus body mass index and diabetes duration.

**Table 6:** Time in ranges according to HGI-FPG in patients with HbA1c ≥ 7.0% and < 7.0%

	HGI-FPG ≤ 0%	HGI-FPG > 0%	P
Patients with HbA1c ≥ 7.0%			
Fasting plasma glucose, mg/dL	165.3 ± 42.1	142.1 ± 33.5	0.010
HbA1c, %	7.7 ± 0.8	8.4 ± 1.2	0.009
Time in range (TIR)	64.0 ± 28.5	65.9 ± 26.4	0.768
Time above range (TAR)	35.0 ± 29.4	32.6 ± 26.9	0.722
Time below range (TBR)	1.0 ± 2.1	1.5 ± 4.2	0.611
Patients with HbA1c < 7.0%			
Fasting plasma glucose, mg/dL	120.1 ± 12.1	81.0 ± 26.5	0.001
HbA1c, %	6.5 ± 0.3	6.6 ± 0.4	0.811
Time in range (TIR)	88.3 ± 10.8	85.6 ± 11.2	0.730
Time above range (TAR)	9.8 ± 11.2	6.8 ± 8.9	0.742
Time below range (TBR)	1.9 ± 2.7	7.6 ± 2.3	0.013

Data are presented as mean ± SD. FPG, fasting plasma glucose. HbA1c, glycated hemoglobin. HGI, hemoglobin glycation index. HGI-FPG, HGI defined as the difference between the observed HbA1c and the predicted HbA1c based on FPG. TIR, the percentage of time that glucose levels are within the target range (70-180 mg/dL). TAR, the percentage of time that glucose levels are above the target range (> 180 mg/dL). TBR, the percentage of time that glucose levels are below the target range (< 70 mg/dL).

## Abbreviations

CGM: continuous glucose monitoring
FPG: fasting plasma glucose
HbA1c: glycated hemoglobin
GMI: glucose management indicator
HGI: hemoglobin glycation index
MAGE: mean amplitude of glycemic excursions
T2D: type 2 diabetes
TIR: time in range
TAR: time above range
TBR: time below range
